# The missing link in gravitational-wave astronomy: A summary of discoveries waiting in the decihertz range

**DOI:** 10.1007/s10686-021-09713-z

**Published:** 2021-04-29

**Authors:** Manuel Arca Sedda, Christopher P. L. Berry, Karan Jani, Pau Amaro-Seoane, Pierre Auclair, Jonathon Baird, Tessa Baker, Emanuele Berti, Katelyn Breivik, Chiara Caprini, Xian Chen, Daniela Doneva, Jose M. Ezquiaga, K. E. Saavik Ford, Michael L. Katz, Shimon Kolkowitz, Barry McKernan, Guido Mueller, Germano Nardini, Igor Pikovski, Surjeet Rajendran, Alberto Sesana, Lijing Shao, Nicola Tamanini, Niels Warburton, Helvi Witek, Kaze Wong, Michael Zevin

**Affiliations:** 1 Astronomisches Rechen-Institut, Zentrüm für Astronomie, Universität Heidelberg, Mönchofstr. 12-14, Heidelberg, Germany; 2 Center for Interdisciplinary Exploration and Research in Astrophysics (CIERA), Department of Physics and Astronomy, Northwestern University, 2145 Sheridan Road, Evanston, IL 60208 USA; 3 SUPA, School of Physics and Astronomy, University of Glasgow, Glasgow, G12 8QQ UK; 4 Department of Physics and Astronomy, Vanderbilt University, Nashville, TN 37212 USA; 5 Universitat Politècnica de València, IGIC, Valencia, Spain; 6 Kavli Institute for Astronomy and Astrophysics, Peking University, Beijing, 100871 China; 7 Institute of Applied Mathematics, Academy of Mathematics and Systems Science, CAS, Beijing, 100190 China; 8 Zentrum für Astronomie und Astrophysik, TU Berlin, Hardenbergstraße 36, 10623 Berlin, Germany; 9 Laboratoire Astroparticule et Cosmologie, CNRS UMR 7164, Université Paris-Diderot, 10 rue Alice Domon et Léonie Duquet, 75013 Paris, France; 10 High Energy Physics Group, Physics Department, Imperial College London, Blackett Laboratory, Prince Consort Road, London, SW7 2BW UK; 11 School of Physics and Astronomy, Queen Mary University of London, Mile End Road, London, E1 4NS UK; 12 Department of Physics and Astronomy, Johns Hopkins University, 3400 N. Charles Street, Baltimore, MD 21218 USA; 13 Canadian Institute for Theoretical Astrophysics, University of Toronto, 60 St. George Street, Toronto, Ontario M5S 1A7 Canada; 14 Astronomy Department, School of Physics, Peking University, Beijing, 100871 China; 15 Theoretical Astrophysics, Eberhard Karls University of Tübingen, Tübingen, 72076 Germany; 16 Kavli Institute for Cosmological Physics, Enrico Fermi Institute, The University of Chicago, Chicago, IL 60637 USA; 17 City University of New York-BMCC, Chambers St, New York, NY 10007 USA; 18 Department of Astrophysics, American Museum of Natural History, New York, NY 10028 USA; 19 Department of Physics, University of Wisconsin – Madison, Madison, WI 53706 USA; 20 Department of Physics, University of Florida, PO Box 118440, Gainesville, Florida 32611 USA; 21 Faculty of Science and Technology, University of Stavanger, 4036 Stavanger, Norway; 22 Department of Physics, Stevens Institute of Technology, Hoboken, NJ 07030 USA; 23 Department of Physics, Stockholm University, SE-10691 Stockholm, Sweden; 24 Università di Milano Bicocca, Dipartimento di Fisica G. Occhialini, Piazza della Scienza 3, I-20126 Milano, Italy; 25 National Astronomical Observatories, Chinese Academy of Sciences, Beijing, 100012 China; 26 Max-Planck-Institut für Gravitationsphysik (Albert-Einstein-Institut), Am Mühlenberg 1, 14476 Potsdam-Golm, Germany; 27 School of Mathematics and Statistics, University College Dublin, Belfield, Dublin 4 Ireland; 28 Department of Physics, King’s College London, Strand, London WC2R 2LS UK

**Keywords:** Gravitational waves, Decihertz observatories, Multiband gravitational-wave astronomy, Multimessenger astronomy, Space-based detectors, Black holes, Neutron stars, White dwarfs, Stochastic backgrounds, Binary evolution, Intermediate-mass black holes, Tests of general relativity, Voyage 2050

## Abstract

Since 2015 the gravitational-wave observations of LIGO and Virgo have transformed our understanding of compact-object binaries. In the years to come, ground-based gravitational-wave observatories such as LIGO, Virgo, and their successors will increase in sensitivity, discovering thousands of stellar-mass binaries. In the 2030s, the space-based *LISA* will provide gravitational-wave observations of massive black holes binaries. Between the $\sim 10$–10^3^ Hz band of ground-based observatories and the $\sim 10^{-4}$–10^− 1^ Hz band of *LISA* lies the uncharted decihertz gravitational-wave band. We propose a *Decihertz Observatory* to study this frequency range, and to complement observations made by other detectors. Decihertz observatories are well suited to observation of intermediate-mass ($\sim 10^{2}$–10^4^*M*_⊙_) black holes; they will be able to detect stellar-mass binaries days to years before they merge, providing early warning of nearby binary neutron star mergers and measurements of the eccentricity of binary black holes, and they will enable new tests of general relativity and the Standard Model of particle physics. Here we summarise how a Decihertz Observatory could provide unique insights into how black holes form and evolve across cosmic time, improve prospects for both multimessenger astronomy and multiband gravitational-wave astronomy, and enable new probes of gravity, particle physics and cosmology.

## The gravitational-wave spectrum

When new frequency ranges of the electromagnetic spectrum became open to astronomy, our understanding of the Universe expanded as we gained fresh insights and discovered new phenomena [1]. Equivalent breakthroughs are awaiting gravitational-wave (GW) astronomy [2, 3]. Here, we summarise the *scientific potential of exploring the* ${\sim 0.01}$–1Hz *GW spectrum*.

The first observation of a GW signal was made in 2015 by the Laser Interferometer Gravitational-Wave Observatory (LIGO) [3]. Ground-based detectors such as LIGO [4], Virgo [5], and KAGRA [6] observe over a frequency spectrum $\sim 10$–10^3^ Hz. This is well tailored to the detection of merging stellar-mass black hole (BH) and neutron star (NS) binaries [7]. Next-generation ground-based detectors, like Cosmic Explorer [8] or the Einstein Telescope [9, 10] may observe down to a few hertz. Only a small part of the GW spectrum can thus be observed by ground-based detectors, and extending to lower frequencies requires space-based observatories.

Lower frequency GW signals originate from coalescences of more massive binaries, and stellar-mass binaries earlier in their inspirals. Due for launch in 2034, the *Laser Interferometer Space Antenna* (*LISA*) will observe across frequencies $\sim 10^{-4}$–10^− 1^ Hz [11], optimal for mergers of binaries with $\sim 10^{6} M_{\odot }$ massive BHs [12–14]. *LISA* will be able to observe nearby stellar-mass binary BHs (BBHs) years–days prior to merger [15], when they could be observed by ground-based detectors. *Multiband* observations of BBHs would provide improved measurements of source properties [16–18], new constraints on their formation channels [19, 20], and enable precision tests of general relativity (GR) [16, 21].

Pulsar timing arrays are sensitive to even lower frequency GWs of $\sim 10^{-9}$–10^− 7^ Hz [22], permitting observation of $\sim 10^{9} M_{\odot }$ supermassive BHs [23]. Combining *LISA* and pulsar timing observations will produce new insights into the evolution of (super)massive BHs [24, 25].

The case for extending the accessible GW spectrum with an observatory that can observe in the $\sim 0.01$–1 Hz *decihertz* range is explained in [26], based upon a White Paper that we submitted in response to ESA’s Voyage 2050 call, and here we summarise the highlights. Decihertz observations would:

1. Reveal the formation channels of stellar-mass binaries, complementing ground-based observations with deep multiband observations.
2. Complete our census of the population of BHs by enabling unrivaled measurements of intermediate-mass BHs (IMBHs), which may be the missing step in the evolution of (super)massive BHs.
3. Provide a new laboratory for tests of fundamental physics.

Decihertz observatories (DOs) have the capability to resolve outstanding questions about the intricate physics of binary stellar evolution, the formation of astrophysical BHs at all scales across cosmic time, and whether extensions to GR or the Standard Model of particle physics are required.

## The potential of decihertz observatories

Decihertz observations would bridge space-based low-frequency detectors and ground-based detectors, giving us access to a wide variety of astrophysical systems:

1. *Stellar-mass binaries comprised of compact stellar objects—white dwarfs (WDs), NSs, and stellar-mass BHs.* Since BH and NS mergers are observable with ground-based detectors, a DO would allow multiband observations of these populations. WDs are inaccessible to ground-based detectors [27], and so can only be studied with space-based detectors. While the current-generation of ground-based detectors will detect stellar-mass BBHs to redshifts $z \sim 1$–2, next-generation detectors will discover them out to $z \sim 20$, enabling them to chart the evolution of the binary population across the history of the Universe [28]; a DO could match this range, far surpassing *LISA*. Furthermore, decihertz observations of compact-object mergers would provide valuable forewarning of multimessenger emission associated with merger events. If detected, multimessenger observations reveal details about the equation of state of nuclear density matter [29–33], the production of heavy elements [34–38], and provide a unique laboratory for testing gravity [39–42], as well as potentially identifying the progenitors of Type Ia supernovae [43–45]. Even without finding a counterpart, correlation with galaxy catalogues can provide *standard siren* cosmological measurements [46–53]. Following their detection by LIGO and Virgo, BHs and NSs are a *guaranteed* class of GW source [3, 7, 54]. With a large number of observations, we can infer the formation channels for compact-object binaries, and the physics that governs them [28, 55–62]. Eccentricity is a strong indicator of formation mechanism [19, 20, 63–66]; however, residual eccentricity is expected to be small in the regime observable with ground-based detectors [67–70] while in some cases, BBHs formed with the highest eccentricities will emit GWs of frequencies too high for *LISA* [65, 71–76]. Hence DOs could provide unique insights into binary evolution.
2. *IMBHs of *$\sim 10^{2}$*–10*^4^*M*_⊙_. IMBHs could be formed via repeated mergers of stars and compact stellar remnants in dense star clusters [77–80]. Using GWs, IMBHs could be observed in a binary with a compact stellar remnant as an intermediate mass-ratio inspiral (IMRI) [81–84], or in a coalescing binary with another IMBH. A DO would enhance the prospects of IMRI detection to tens of events per year, with observations extending out to high redshift. Mergers involving a WD or a NS can lead to tidal disruption events with a bright electromagnetic counterpart [85, 86]. IMBHs binaries could be studied across the entire history of the Universe, charting the properties of this population and constraining the upper end of the pair-instability mass gap [87], while also providing a detailed picture of the connection (or lack thereof) between IMBHs and the seeds of massive black holes [88]. The connection between massive BHs and their lower-mass counterparts could be further explored through observations of binaries orbiting massive BHs in galactic centres, or around IMBHs in smaller clusters [76, 89–97]. BBH–IMBH systems are a target for DO–ground-based multiband observation because they emit both 1–10^2^ Hz GWs and *simultaneously* 0.01–1 Hz GWs.
3. *Cosmological sources as part of a stochastic GW background (SGWB).* Both this and the other astrophysical sources serve as probes of new physics, enabling tests of deviations from GR and the Standard Model. A first-order phase transition in the early Universe can generate a SGWB [98–103]; a DO would be sensitive to first-order phase transitions occurring at higher temperature, or with a shorter duration, compared to *LISA*. A DO would be sensitive to a SGWB from a source at $\sim 1~\text {TeV}$ and beyond: TeV-scale phenomena have been consider to resolve with the hierarchy problem or the question of dark matter [104–109], while 100 TeV-scale phenomena appear in new solutions to the hierarchy problem such as the relaxion [110, 111]. Furthermore, SGWB (non-)detection could constrain the properties of cosmic strings [112, 113] down to string tensions of $\sim 10^{-19}$, while *LISA* would reach $\sim 10^{-17}$ [114] and pulsar timing array observations currently constrain tensions to be $\lesssim 10^{-11}$ [115, 116].

Decihertz observations provide a unique insight into the physics of each of these sources, and observations would answer questions on diverse topics ranging from the dynamics of globular clusters to the nature of dark matter.

## Decihertz mission concepts

**Fig. 1:**
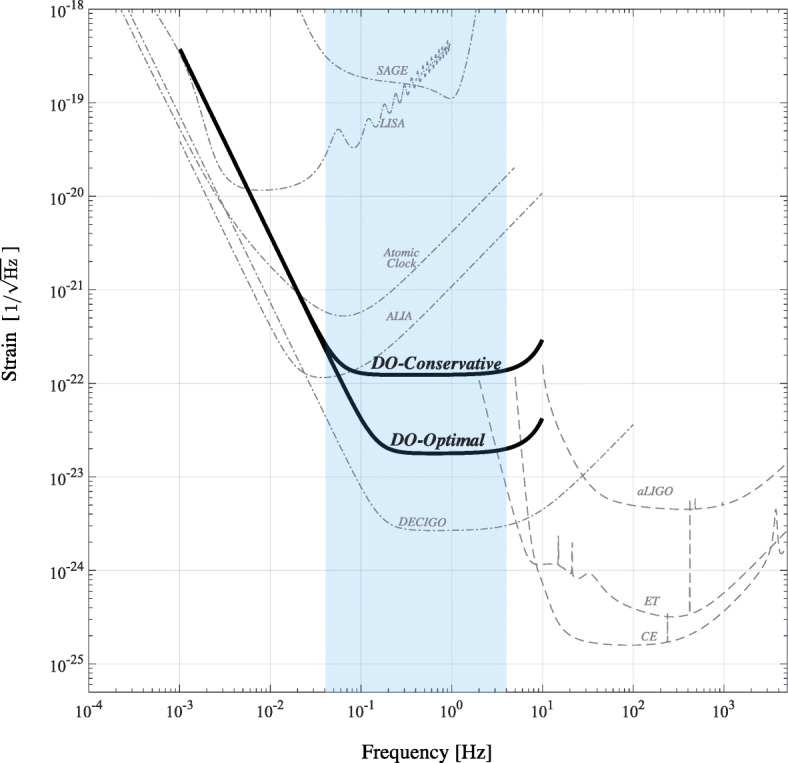
Concept designs for Decihertz Observatories (DOs) fill the gap between *LISA* [11] and ground-based detectors like Advanced LIGO (aLIGO) [4], Cosmic Explorer (CE) [8] and the Einstein Telescope (ET) [10]. The example DO concepts *SAGE* [129, 130], Atomic Clock [26, 131], *ALIA* [117, 118], DO-Conservative, DO-Optimal [26, 135] and *DECIGO* [123, 124] span a diverse set of technologies and illustrate the potential range in sensitivities

The scientific return of a DO will depend upon its design. There are multiple potential technologies and mission concepts for observing the 0.01–1 Hz GW spectrum. The *Advanced Laser Interferometer Antenna* (*ALIA*) [117, 118] is a heliocentric mission concept more sensitive than *LISA* in the 0.1–1 Hz range. Other heliocentric DO concepts are *Taiji* [119, 120], most sensitive around 0.01 Hz, and *TianGo* [121], most sensitive in the 0.1–10 Hz range. *TianQin* [122] is a Chinese geocentric mission concept. The *DECi-hertz Interferometer Gravitational-wave Observatory* (*DECIGO*) [123, 124] is a more ambitious concept with 1000 km Fabry–Perot cavity arms in heliocentric orbit; its precursor *B-DECIGO* would be a 100 km triangular interferometer in a geocentric orbit. The *Big Bang Observer* (*BBO*) is a concept consisting of four *LISA* detectors in heliocentric orbits with combined peak sensitivity over 0.1–1 Hz range [125]. More modest designs are the *Geostationary Antenna for Disturbance-Free Laser Interferometry* (*GADFLI*) [126] and *geosynchronous Laser Interferometer Space Antenna* (*gLISA*) [127, 128] which are geocentric concepts. The *SagnAc interferometer for Gravitational wavE* (*SAGE*) [129, 130] consists of three identical CubeSats in geosynchronous orbit. These concepts are mostly variations on the classic *LISA* design of a laser interferometer. In addition to laser interferometry, atomic-clock-based [131, 132] and atom interferometer concepts are in development; for example, the *Mid-band Atomic Gravitational Wave Interferometric Sensor* (*MAGIS*) [133] and the *Atomic Experiment for Dark Matter and Gravity Exploration in Space* (*AEDGE*) [134] designs use atom interferometry. The range of technologies available mean that there are multiple possibilities for obtaining the necessary sensitivity in the decihertz range. Two illustrative *LISA*-like designs, the more ambitious DO-Optimal and the less challenging DO-Conservative, are presented in [26] to assess the potential range of science achievable with DOs. Potential sensitivity of DOs are illustrated in Fig. 1 in comparison to other gravitational-wave observatories.

## Summary

Observing GWs in the decihertz range presents huge opportunities for advancing our understanding of both astrophysics and fundamental physics. The only prospect for decihertz observations is a space-based DO. Realising the rewards of these observations will require development of new detectors beyond *LISA*. *There are many challenges in meeting the requirements of DO concepts; however, there are also many promising technologies that could be developed to meet these goals*. A DO mission ready for launch in 2035–2050 is achievable, and the science payoff is worth the experimental effort.
